# Increased Infarct Wall Thickness by a Bio-Inert Material Is Insufficient to Prevent Negative Left Ventricular Remodeling after Myocardial Infarction

**DOI:** 10.1371/journal.pone.0021571

**Published:** 2011-06-23

**Authors:** Aboli A. Rane, Joyce S. Chuang, Amul Shah, Diane P. Hu, Nancy D. Dalton, Yusu Gu, Kirk L. Peterson, Jeffrey H. Omens, Karen L. Christman

**Affiliations:** 1 Department of Bioengineering, University of California San Diego, La Jolla, California, United States of America; 2 Department of Medicine, University of California San Diego, San Diego, California, United States of America; University of California Merced, United States of America

## Abstract

**Background:**

Several injectable materials have been shown to preserve or improve cardiac function as well as prevent or slow left ventricular (LV) remodeling post-myocardial infarction (MI). However, it is unclear as to whether it is the structural support or the bioactivity of these polymers that lead to beneficial effects. Herein, we examine how passive structural enhancement of the LV wall by an increase in wall thickness affects cardiac function post-MI using a bio-inert, non-degradable synthetic polymer in an effort to better understand the mechanisms by which injectable materials affect LV remodeling.

**Methods and Results:**

Poly(ethylene glycol) (PEG) gels of storage modulus G′ = 0.5±0.1 kPa were injected and polymerized *in situ* one week after total occlusion of the left coronary artery in female Sprague Dawley rats. The animals were imaged using magnetic resonance imaging (MRI) at 7±1 day(s) post-MI as a baseline and again post-injection 49±4 days after MI. Infarct wall thickness was statistically increased in PEG gel injected vs. control animals (*p*<0.01). However, animals in the polymer and control groups showed decreases in cardiac function in terms of end diastolic volume, end systolic volume and ejection fraction compared to baseline (*p*<0.01). The cellular response to injection was also similar in both groups.

**Conclusion:**

The results of this study demonstrate that passive structural reinforcement alone was insufficient to prevent post-MI remodeling, suggesting that bioactivity and/or cell infiltration due to degradation of injectable materials are likely playing a key role in the preservation of cardiac function, thus providing a deeper understanding of the influencing properties of biomaterials necessary to prevent post-MI negative remodeling.

## Introduction

Progressive left ventricular (LV) remodeling occurs after myocardial infarction (MI), and while remodeling can be beneficial initially, over time it can become maladaptive leading to LV dysfunction [1]. With 5.8 million people suffering from heart failure (HF), treatment of negative LV remodeling after MI, the leading cause of HF, is a pressing clinical need [2]. Coagulative necrosis following MI initiates a cascade of events characterized by myocyte necrosis and their eventual replacement by fibrotic scar tissue. Necrosis of the myocytes triggers an inflammatory response leading to activation of matrix metalloproteinases [3] that cause a breakdown of the extracellular matrix, which results in myocyte slippage and infarct expansion. At the same time fibroblast proliferation occurs, leading to deposition of collagen and the formation of scar tissue. Concurrent TGF-β1 mediated transformation of fibroblast to myofibroblasts leads to deposition of type I and III collagen and scar tissue formation from subsequent collagen cross-linking [1]. Ventricular wall thinning and dilatation also occurs leading to an increase in wall stress, and ultimately resulting in heart failure [1], [4], [5].

Various injectable biomaterials have been investigated as potential acellular treatments to prevent or reverse this downward spiral of adverse LV remodeling [6]. Collagen [7], fibrin [8], chitosan [9], small intestinal submuscosa (SIS) [10], and a collagen-matrigel blend [11] as well as a few degradable synthetic materials [12], [13], [14] have shown either an improvement or maintenance of cardiac function in terms of ejection fraction or fractional shortening. Injection of alginate has been shown to cause a decrease in LV diastolic area and systolic area [15], [16] while fibrin [17], hyaluronic acid (HA) [18], SIS [10] and a synthetic thermoresponsive polymer [14] reduced infarct size. Injection of biomaterials such as collagen [19], matrigel [19], fibrin [17], [19], alginate [20], chitosan [9], self-assembling nanofibers [21], SIS [10], and a degradable thermoresponsive polymer [13] have also increased neovasculature. Though most injectable materials have shown positive effects on cardiac function and LV remodeling, to date, the mechanisms behind this improvement are unknown.

It has been shown that myocardial infarction can lead to an increase in wall stress due to changes in wall curvature and thickness in accordance with Laplace's Law, which states that wall stress is directly proportional to the pressure and radius, and inversely proportional to thickness. Wall thinning due to infarction can increase wall stress, and may contribute to border zone expansion and progression of negative LV remodeling [22]. Hence, it has been hypothesized that injectable materials may affect the passive structural properties of the wall by increasing infarct wall thickness and hence reducing local LV wall stress [6], [23]. This decrease in LV wall stress is thought to protect the vulnerable myocardium from stress induced apoptosis and infarct expansion, thus preventing pathological LV remodeling and decline in cardiac function [23]. On the other hand, injectable materials have also been shown to elicit cell infiltration [19], [21], [24] and neovascularization [19] in the affected area. This leads to the alternate hypothesis that the bioactivity of materials, such as inherent cell adhesion domains or angiogenic degradation products, and/or cell infiltration upon degradation of these materials are playing an important role in prevention of adverse LV remodeling and improvement of cardiac function [6], [25], [26].

The aim of this study was to begin to understand the mechanisms by which injectable materials preserve cardiac function and prevent negative LV remodeling post-MI by decoupling bioactivity from the structural effects of an injectable polymer. Herein, we utilized an injectable, bio-inert, non-degradable polymer, polyethylene glycol (PEG), along with experimental parameters similar to those employed in previous studies aimed at assessing the effects of injectable biomaterials on LV remodeling and cardiac function. PEG is a well established synthetic polymer that is known to be non-toxic [27] and bio-inert, thus preventing protein and cell adhesion [27], [28], [29], [30]. We demonstrate that passive structural intramyocardial support by itself does not prevent negative LV remodeling or maintain cardiac function, suggesting that other mechanisms such as bioactivity and/or cell infiltration seen with degradable materials likely play a dominant role in the mitigation of LV remodeling and preservation of cardiac function.

## Materials and Methods

All experiments in this study were conducted in accordance with the guidelines established by the Institutional Animal Care and Use Committee at the University of California, San Diego and the American Association for Accreditation of Laboratory Animal Care and were approved by the Institutional Animal Care and Use Committee at UCSD (A3033-01). All efforts were made to minimize any pain and suffering felt by the animals.

### PEG hydrogel preparation

PEG hydrogels were prepared by mixing solutions of 4-arm polyethylene glycol-amide-succinimidyl glutarate (PEG-ASG) (Sunbio, Anyang City, South Korea, MW 20,000) and trilysine (Sigma-Aldrich, St. Louis, MO) to create PEG hydrogels of desired stiffness at room temperature by chemical crosslinking [31]. Optimal concentrations of PEG-ASG and trilysine were experimentally determined to generate gels of stiffness similar to that of commonly injected biomaterials. Equal volumes of 100 mg/ml PEG-ASG in pH 4.0 phosphate buffer and 0.5–2 mg/ml trilysine in pH 8.2 borate buffer were mixed together to prepare the hydrogels. For *in-vivo* injections of PEG, the solutions were mixed together and injected prior to gelation.

### Mechanical testing

Material properties in terms of the storage modulus (G′) and loss modulus (G″) were determined using a parallel plate rheometer (TA Instruments AR-2000) as previously reported [32], [33]. Briefly, gels were placed between two parallel plates of the rheometer and compressed to a normal force between 0.2–0.3 N, to avoid slipping. A strain sweep (0.0001 to 10%) was performed at a constant frequency of 1 Hz to confirm that the measurements were within the linear viscoelastic region. 1% constant strain was used for subsequent frequency sweeps (0.01 to 10 Hz) using a logarithmic scale, taking 5 points per decade. All measurements were taken in triplicate and reported as mean ± SEM at a frequency of 1 Hz.

### Rat total occlusion model

Left coronary artery total occlusion was performed on female Sprague Dawley rats (225–250 g) under aseptic conditions. Animals were anesthetized using 5% isoflurane, intubated and maintained at 2.5% isoflurane for the surgical procedure. The animals were ventilated using a respirator at 75 breaths/minute. The heart was exposed using a left anterior thoracotomy, and the artery was ligated using a 6-0 silk suture at 1–2 mm below the left atrial appendage as previously reported [34]. The chest was closed and the animals were allowed to recover. ECG was continuously monitored for detection of arrhythmias, and atropine 150 to 200 µl (0.54 mg/ml) was administered I.P. if needed. Buprenorphine was administered I.P. at 5 mg/kg to prevent post-operative pain and Ringer's Lactate (3 cc) was given I.P. to the animals to prevent dehydration.

### Echocardiography

Echocardiography was performed 4±1 day(s) post-MI to screen for the presence of an infarct. Animals were anesthetized using 5% isoflurane for 30 seconds and then maintained at 1% isoflurane for the imaging procedure. Parasternal long axis and short axis images were obtained using a Philips, Sonos 5500 system with a 15 MHz transducer. Qualitative assessment of infarction size was rendered at the time of imaging using both planes. Animals with either no MI or very small MI (less than 15% of the LV perimeter in the long axis plane) were excluded from the study prior to injections.

### Injection surgery

The animals were randomized 9±1 day(s) after MI, and injected with either 100 µl of PEG hydrogel or saline (control). An incision was made between the fourth and fifth ribs, the anterolateral portion of the heart was exposed, and an injection of polymer or saline was administered using a 27 G needle into the infarct wall. Presence of the injection was verified by temporary discoloration of the tissue. The chest was then closed and the animal was allowed to recover. To prevent post-operative pain in the animals, buprenorphine was administered at 5 mg/kg.

### Magnetic resonance imaging

Magnetic Resonance (MR) imaging was used for quantitative assessment of the injected gel on cardiac function. Animals were scanned at 7±1 day(s) post-MI, as a baseline measurement, and again at 49±4 days after MI to assess post-treatment effects on remodeling and cardiac function. A 7T Bruker Horizontal Bore scanner and Bruker single tuned quadrature Transmit/Receive volume coil were used for all measurements. Anatomical cine imaging was performed with an ECG-gated Fast Low Angle Shot (FLASH) sequence as previously reported [35]. MR parameters were set to TR = 7.7 ms, TE = 1.28 ms, flip angle = 15° and 4–6 averages per slice. All slices were 1 mm in thickness, had a field of view of 50 mm×50 mm and were contained in a data matrix of 256×256 pixels. For each slice, 25 frames were taken through the cardiac cycle to capture end diastolic and end systolic images. Animals were anesthetized using 1.5–2% isoflurane and the heart rate, ECG and respiration rate were monitored continuously. The long axis of the heart was identified and 10–12 short axis slices from apex to base were acquired at 1 mm increments. ImageJ was used to trace the endocardial boundary at end diastole (ED) and end systole (ES) on each short axis slice. Simpson's method was used to determine the end diastolic volume (EDV) and end systolic volume (ESV). Ejection fraction (EF) was calculated as [(EDV-ESV)/EDV]×100 similar to previous methods [36].

### Finite element modeling

In order to visually represent LV dilatation and wall thickness pre and post-injection, finite element analysis was used to generate geometric models of the LV at ED and ES using prolate spheroidal bi-cubic Hermite finite element surface meshes [35]. Meshes were fit to the endocardial and epicardial boundary points determined from the MRI cine images. The boundary points were fit using a least squares minimization in the λ coordinate direction with bicubic Hermite interpolation. The 40 element mesh was refined to 340 elements and was converted to Cartesian coordinates so that the long axis of the heart aligned with the X- axis and the septum was bisected by the Y-axis.

### Histology and immunohistochemisty

Animals were sacrificed 24 hours after post-treatment MR imaging (∼49 days post-MI) by an IP injection of Fatal Plus (sodium pentobarbital, 936 mg/kg). Hearts were immediately excised and fresh frozen in Tissue-Tek O.C.T. freezing compound. Hearts were sectioned into 10 µm slices and slides taken every 0.4 mm were stained with hematoxylin and eosin (H&E) to visualize the polymer injection. Images were taken using a Carl Zeiss Observer D.1 and analyzed using AxioVision software. A pathologist qualitatively assessed inflammatory response [37], [38], [39] to the injection in the infarct, border zone and remote tissue in the H&E stained sections at mid-infarct. Macrophages were identified using an anti-rat CD68 primary antibody [40]. Tissue sections were incubated with 1∶50 dilution of mouse anti-rat CD68 and subsequently incubated with horseradish peroxidase (HRP) conjugated goat anti-mouse IgG (1∶100 dilution). The HRP was visualized by incubation with diaminobenzidine (DAB) for 10 minutes. Wall thickness was calculated using 3 mid-infarct slides, averaging 5 equally spaced measurements along the infarct wall in each slide. Infarct size was calculated using H&E stained sections by calculating the average of the ratio of the endocardial infarct length to the endocardial circumference and the ratio of the epicardial infarct length to the epicardial circumference over three slides evenly spaced through the infarct as previously shown [41]. To assess arteriole density, immunohistochemistry staining was done on three slides evenly spaced through the polymer region or infarct for each of the PEG and saline injected hearts respectively, according to previously described methods [17], [20], [24]. Briefly, the sections were fixed with acetone, incubated with anti-smooth muscle actin antibody and then stained with Alexa Fluor 568 anti-mouse antibody. Vessels in the range of 10 µm to 100 µm in the infarct were quantified with Axiovision.

### Statistical analysis

Two-tailed Students' t-test was used for infarct wall thickness and arteriole density analyses. Differences between baseline and post-treatment groups with MRI analysis were assessed using a paired two-tailed t-test. All measurements were reported mean ± SEM. Significance was accepted at *p*<0.05.

## Results

### PEG hydrogel mechanical characterization

**Figure 1:**
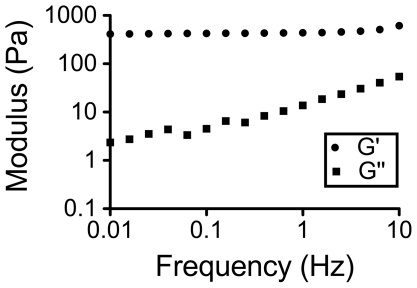
Mechanical spectra. Typical mechanical spectra for a hydrogel formed by crosslinking of 4-arm PEG-ASG (100 mg/ml) and trilysine (1 mg/ml).

To create a PEG hydrogel with desired mechanical properties, the amount of trilysine crosslinker (0.5 mg/ml to 2 mg/ml) was varied. A strain sweep test and a frequency sweep test were performed to generate mechanical spectra of the gels. We sought to test a polymer with similar mechanical properties to commonly injected materials to better delineate between structural effects and bioactivity. A storage modulus (G′) of 0.5±0.1 kPa (trilysine 1 mg/ml) (Figure 1) was selected as it mimicked the mechanical properties of commonly injected polymers [42], [43]. As expected the G′ is independent of frequency and G″ (loss modulus) is weakly dependent on frequency [33], [44].

### LV geometry and cardiac function

*In-vivo* studies were performed to assess the effect of the bio-inert, non-degradable PEG hydrogel on wall thickness, EF, EDV and ESV. All animals underwent a surgery to induce MI. After screening echocardiography, 17 out of 33 animals were included in the study based on presence of a sizable infarct. One additional animal died during intubation prior to injection. Sixteen animals therefore underwent either polymer (n = 8) or saline (n = 8) injections. 100% of the animals survived the injection surgery; however, 3 animals were removed from functional analysis, 2 animals from the saline group and 1 from the polymer group, due to inconsistent gating during MR imaging. These animals were included in the histological analysis. One animal from the polymer group was excluded from the entire study due to insufficient polymer injection based on histology. In addition, infarct size was not different in the PEG (44.1%±2.8%) and saline (48.0%±2.8%) injected animals (p = 0.336).

**Figure 2:**
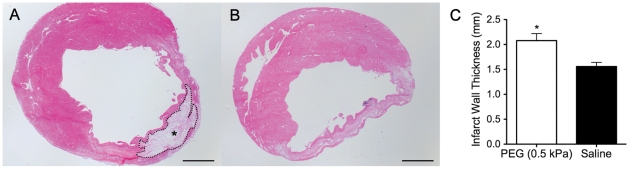
Histological assessment. Histological assessment of polymer and saline injections 7 weeks after MI. Representative slides stained with H&E: (A) PEG hydrogel (G′ = 0.5 kPa) injected in infarct region and (B) saline injected in infarct region. (C) Infarct wall thickness at 7 weeks post-MI. Wall thinning was prevented in the PEG injected group compared to the control. (*p<0.01, PEG: n = 7, saline: n = 8 ) Photomicrographs are taken at 10Х and scale bars are 1 mm. PEG area outlined with dotted line and demarcated by an asterisk (*).

**Figure 3:**
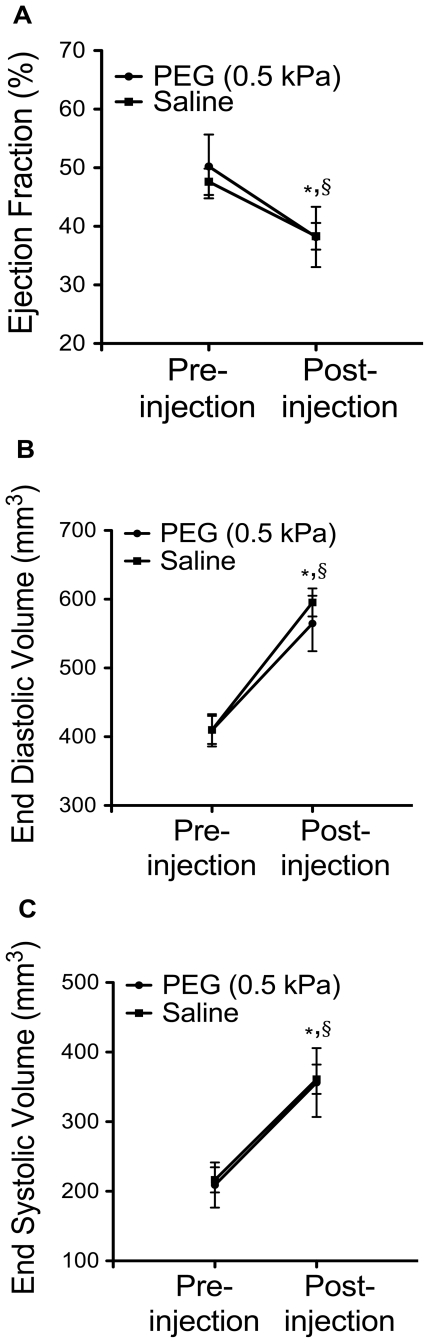
Cardiac function. Comparison of cardiac function baseline (1 week post-MI, pre-injection) and post-treatment (7 weeks post-MI) in PEG (0.5 kPa) injected and saline injected groups. (A) Ejection fraction. (B) End diastolic volume. (C) End systolic volume. Statistical significance determined using paired two-tailed *t*-test between pre and post-injection groups. (*p<0.01 PEG compared to baseline, § *p*<0.01 saline compared to baseline, PEG: n = 6, saline: n = 6).

Animals injected with the PEG hydrogel showed a significantly thicker infarct wall in comparison to saline injected animals (p = 0.006) as seen via histological analysis (Figure 2). Despite the increase in wall thickness, analysis of the MR images showed a steady decline in cardiac function and expansion of LV volume by comparing values pre- and post-treatment (Figure 3). At 7 weeks there was a significant increase in LV EDV (p = 0.0016) and ESV (p = 0.0008), as well as a decrease in EF (p = 0.006) in the polymer group. As expected in the saline control group, LV EDV (p = 0.000002) and ESV (p = 0.0001) significantly expanded with a corresponding decline in EF (p = 0.005). The heart rate of the animals was continuously monitored during the imaging process and was statistically similar between the PEG and saline injected groups: 278±17 beats/min (PEG pre-injection) and 281±8 beats/min (PEG post-injection); 271±14 beats/min (saline pre-injection) and 285±8 beats/min (saline post-injection). The ECG was carefully monitored during the time of image acquisition. There were no noticeable arrhythmias.

**Figure 4:**
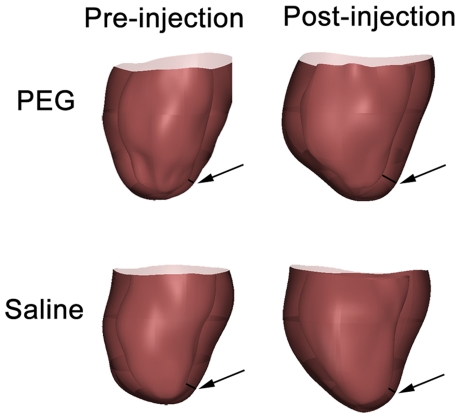
3-D representation of the LV. Finite element representation of the LV pre and post-injection in both PEG and saline injected groups. Arrow denotes region of infarction. Infarct wall thickening is seen in the PEG injected heart while infarct wall thinning is depicted in the saline injected heart compared to baseline. Also note the dilation of LV lumen pre and post-injection in both groups.

As a 3-D visual representation of the overall effect of polymer injection, finite element models were generated using the endocardial and epicardial boundaries traced from the MR images in ED (Figure 4). The overall change in infarcted LV free wall thickness is seen pre- and post-injection (Figure 4, arrows). The hearts injected with the PEG hydrogel demonstrated a regional increase in wall thickness in the infarcted area while the saline injected hearts showed a thinning of the infarct region at 7 weeks. Dilation of ventricle is clearly depicted by enlargement of the LV lumen at 7 weeks as compared to baseline.

### Cellular response to injection

**Figure 5:**
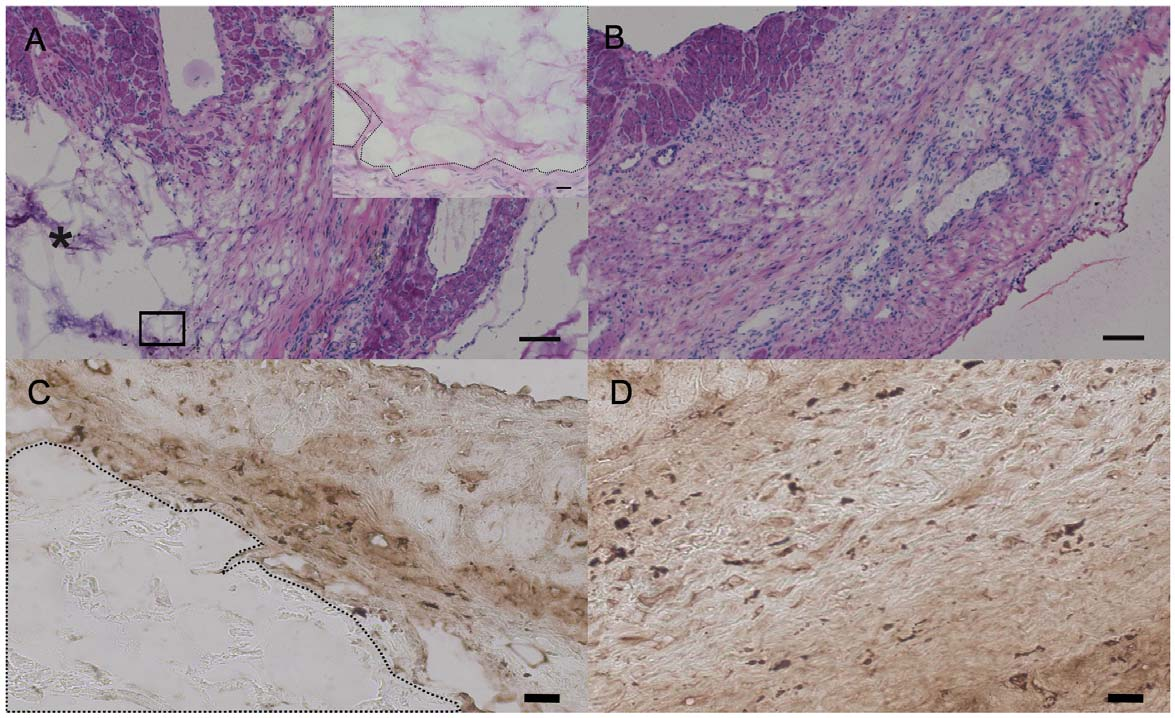
Inflammatory response. Inflammatory response in polymer and saline injected hearts 7 weeks after MI. (A) PEG injected heart (inset shows high magnification image of PEG region showing no cell infiltration in the polymer, scale bar 20 µm) (B) saline injected heart. Scale bars are 100 µm. PEG area is demarcated by an asterisk (*). CD68 stained macrophages in (C) PEG injected heart (D) saline injected heart. PEG area outlined with a dotted line. Scale bars are 20 µm.

**Figure 6:**
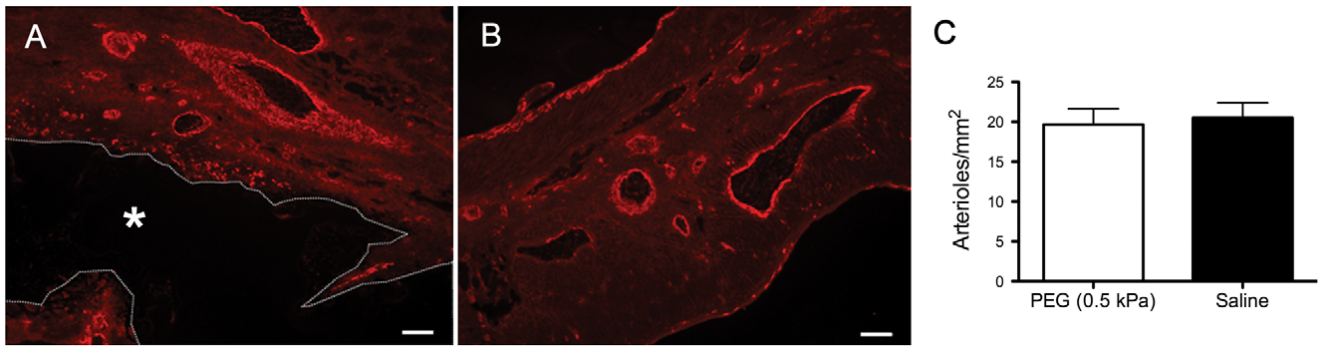
Arteriole density. Arteriole density assessment in polymer and saline injected hearts 7 weeks after MI. (A, B) Representative slides with staining for smooth muscle actin. (A) Arterioles in infarct region of PEG injected animals. PEG area outlined with dotted line and demarcated by an asterisk (*). (B) Arterioles in infarct region of saline injected animals. Scale bars are 100 µm. (C) Arteriole density (vessel diameter between 10 µm and 100 µm) at 7 weeks post-MI. ( p = 0.80, PEG: n = 6, saline: n = 8 ).

As expected there was a very thin layer of encapsulation around the PEG injection region; however, there were no apparent differences in the inflammatory response (macrophages, fibroblasts, foreign body giant cells) between the polymer and saline injected animals in the infarct, border zone or remote myocardium as expected due to the bio-inert properties of PEG [27], [28], [29], [30] (Figure 5A & 5B). To further confirm this assessment, we examined the tissue for presence of CD68^+^ macrophages and confirmed that there was no accumulation of macrophages in PEG injected hearts (Figure 5C & 5D). Immunohistochemistry was performed on multiple sections through the hearts to assess the effect of polymer injection on potential neovascularization in the infarct area. There was no significant difference in arteriole density for vessel diameters ranging between 10 µm and 100 µm in the infarct region of the polymer (19±2 arterioles/mm^2^) and saline (20±2 arterioles/mm^2^) injected hearts, or when vessels were binned as <10 µm, between 10 µm and 25 µm, between 25 µm and 50 µm and between 50 µm and 100 µm, suggesting that polymer injection did not promote increased vessel formation (Figure 6).

## Discussion

To date, the exact mechanism by which injectable materials lead to an improvement in cardiac function is unknown. As most injectable biomaterials cause an increase in infarct wall thickness, some have hypothesized that this passive structural enhancement alone may have therapeutic benefit [6], [23]. After MI, there is typically a thinning of the myocardial wall and replacement with scar tissue. According to the Law of Laplace, this thinning can cause an increase in wall stress that can cause infarct expansion and apoptosis of the myocytes, which in turn can lead to dilation of LV and subsequent HF [4], [23]. Thus, it has been proposed that use of an injectable material to increase wall thickness may lead to a decrease in wall stress and hence prevent adverse LV remodeling [23]. Alternatively, bioactivity or degradation properties of biomaterials may lead to the beneficial effects of these materials in the post-MI setting. The majority of biomaterials that have been examined to date are degradable materials that allow for cell infiltration, as well as biopolymers that have inherent bioactivity. In an effort to begin to understand the mechanisms behind which injectable polymers improve cardiac function, we chose to examine a bio-inert, non-degradable synthetic polymer, PEG, to decouple a material's structural effects from its bioactivity. We chose a compliant gel that mimicked the mechanical properties of commonly injected polymers. Results from this study indicate that injection of PEG leads to an increase in infarct wall thickness, consistent with what has previously been seen with other materials [7], [8], [14], [15]. However, this was insufficient to prevent negative LV remodeling or improve cardiac function as determined through MRI. A strictly structural enhancement of the LV wall may therefore be insufficient for preservation or improvement of cardiac function and/or slowing the progression of negative LV remodeling after MI. These results therefore indicate that other properties of injectable biomaterials may be playing an important role in maintenance or enhancement of function post-MI.

In this study, we show that injection of the non-degradable PEG does not induce post-MI neovascularization, nor does it increase cell infiltration in general. In contrast, many previously injected materials that have shown improvement in cardiac function display increased neovascularization, possibly restoring the blood supply to the affected area and preventing negative LV remodeling [9], [19], [21]. The increase in new vessel formation could be due to bioactive components of the materials such as angiogenesis inducing degradation products (e.g. Fibrin fragment E [45] and peptide fragment hepIII of collagen IV [46]) Unlike PEG, most of these biomaterials also have integrin binding sites that are important for the influx of cells, such as endothelial cells and myofibroblasts [47]. Huang *et al* have shown that injection of collagen in the infarct induced significant myofibroblast infiltration. Similarly there was also a trend for increased myofibroblast migration in fibrin and matrigel injected hearts [19]. It has been shown that the contractile nature of myofibroblasts may lead to infarct size reduction and mitigation of infarct expansion, leading to overall improvement in function [48]. In addition to biopolymers, beneficial effects with a few degradable synthetic polymers that allow for cell influx have also been observed [13]. Therefore, cell infiltration due to degradation of injected polymers might be a potential mechanism by which these materials elicit salutatory effects. Though this effect with biopolymers is likely a result of their inherent bioactivity, these materials have also been shown to degrade in the range of 1–6 weeks [9], [17], [19]. While most of these infiltrating cells cannot restore function to the damaged area, it is possible that the migration of these cells in a timely manner provides beneficial paracrine effects necessary to prevent or lessen infarct scar expansion that is commonly seen post-MI.

While most materials have been degradable, there is one report of a synthetic non-degradable polymer being used for treatment of MI; Dobner *et al* [49], injected a non-degradable PEG in the infarct immediately post-MI and showed that there was temporary retardation of LV remodeling at early time points, but not at later time points. However, the progression of LV remodeling at later time points is aligned with the presented results. It is challenging to make a direct comparison between this study and the presented work as the material properties of the PEG gel were not characterized, and the PEG was injected at a different time point. An additional study has also examined the effects of mechanical properties of materials in an MI setting. Ifkovits *et al* [18], investigated the effect of mechanical modulation of injectable HA hydrogels on LV remodeling and cardiac function in an ovine MI model, finding a smaller infarct area in animals injected with the higher mechanical modulus HA. In that study, the HA spread more interstitially through the infarct, which could have contributed to the differences seen in the results of the presented work and the study performed by Ifkovits *et al*. However, similar to the results presented here, there was a reduction in ejection fraction in all groups compared to baseline. Furthermore, in a study by Garbern *et al*, a pH sensitive, temperature responsive synthetic polymer was injected with or without the addition of bFGF in a rodent infarct model. The control group injected with polymer alone increased infarct wall thickness, yet showed a steady decline in fractional shortening comparable to that of the saline injected control, indicating the polymer injection did not improve cardiac function [50]. While the polymer was designed to degrade, it did not degrade over the course of the study, thus providing additional evidence that degradation may be a key biomaterial parameter for preserving or improving cardiac function post-MI.

In the present study, MRI was the imaging modality of choice. It is well established that MRI is the gold standard for cardiac imaging [51], [52]. Commonly, echocardiography is used for the measurement of cardiac function after injection of a biomaterial. Measurements are made at the level of the papillary muscles that function as an anatomical marker. In the commonly used total occlusion MI model, the anteroapical portion of the heart is most severely affected. The effects of LV dilatation and systolic bulging are thought to be most pronounced in this region. It is possible that by taking measurements at one level the effect of LV dilatation distal to the papillary muscles is overlooked and local effects are being underemphasized. However, using MRI we were able to determine the overall effect of the polymer injection.

As elucidated here, there may be various factors that are potentially contributing to the beneficial effect seen with injectable materials post-MI. In order to better understand the role of biomaterials in MI treatment and compare our findings to previous work, the PEG polymer used in this study was specifically engineered to have properties similar to other previously examined injectable biomaterials. Moreover, we mimicked common study parameters such as the timing of injection and the injection volume. There are however some differences and limitations to our findings. For example, this PEG gels rapidly upon injection limiting the interstitial spread of the material, allowing it to serve as a bulk filler and partial wall replacement. One potential limitation may be the inability of the polymer bolus to distribute through the entire area of the infarct as seen with external LV restraints [53]. This inhomogeneity in spread could lead to regional abnormalities in function as well as allow for infarct expansion in regions where the polymer is not present. Moreover, there could also still be compensatory expansion of the non-infarcted LV that negates any structural support provided by the material injection. In larger animals, multiple injections can ensure more homogeneous spread of material, although 100% coverage of the wall would still not be achievable. Despite the single injection in the majority of rodent experiments, injection of biomaterials have still improved or preserved cardiac function. In addition to injection spread, timing of injection may also be an important parameter. While some may argue at one week post MI the initial expansion of the ventricle has already occurred, most previous studies injected material in the infarct approximately 1 week post-infarction [7], [8], [9], [15], [17], [19], [54]. Injection at this time point also represents greater clinical translatability due to fear of ventricular rupture at earlier time points. In the literature, volumes of 50 µL to 150 µL have been injected in rat hearts [7], [8], [9], [15], [17], [19], [49], [54], [55], [56], [57], hence 100 µL injection volume was selected as it falls in this range and is the volume used most frequently in biomaterial injection studies.

In conclusion, passive structural enhancement by injection of a synthetic, non-degradable polymer into a developing infarct did not improve global cardiac function and was insufficient to alter post-MI remodeling, thereby suggesting that the bioactivity and/or cell infiltration seen with degradable materials may likely play an important role in preserving cardiac function. The results of this study provide additional insight on the important and influencing properties of biomaterials necessary for preventing post-MI negative remodeling. Furthermore, this study highlights the need for further studies that closely examine biomaterial parameters in order to guide the design of enhanced biomaterials therapies for MI.
